# Recurrent esophageal adenocarcinoma derived from ectopic gastric mucosa: A case report

**DOI:** 10.1111/1759-7714.14339

**Published:** 2022-02-01

**Authors:** Nao Kitasaki, Yoichi Hamai, Toru Yoshikawa, Manabu Emi, Tomoaki Kurokawa, Ryosuke Hirohata, Manato Ohsawa, Morihito Okada

**Affiliations:** ^1^ Department of Surgical Oncology Hiroshima University Hiroshima Japan

**Keywords:** adenocarcinoma, cancer, ectopic gastric mucosa, esophagus, inlet patch

## Abstract

Most primary esophageal adenocarcinomas arise from the Barrett epithelium of the distal esophagus. Thus, cancer developing from the ectopic gastric mucosa (EGM) of the proximal esophagus is extremely rare. Furthermore, recurrent adenocarcinoma at the EGM has not been reported. Here, we describe adenocarcinoma originating from the EGM at the boundary of the cervical and thoracic esophagus that recurred twice at the same site within 40 months. This adenocarcinoma was treated throughout its course by three endoscopic submucosal dissections and a subsequent thoracoscopic esophagectomy. This is the first description of recurrent adenocarcinoma originating from the EGM of the proximal esophagus.

## Graphical abstract

We describe adenocarcinoma originating from the ectopic gastric mucosa (EGM) at the boundary of the cervical and thoracic esophagus that recurred twice at the same site within 40 months. This adenocarcinoma was treated throughout its course by three endoscopic submucosal dissections and a subsequent thoracoscopic esophagectomy. This is the first description of recurrent adenocarcinoma originating from the EGM of the proximal esophagus.

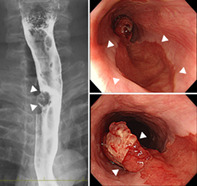

## INTRODUCTION

Ectopic gastric mucosa (EGM) in the esophagus is considered to persist when the esophageal mucosa, which is covered with columnar epithelium at the fetal stage, is replaced by squamous epithelium from the middle esophagus in upward and downward directions.^1^, ^2^ The incidence of EGM in the esophagus is <1–18%, and it mostly occurs in the proximal esophagus.^2^, ^3^ Esophageal EGM is usually asymptomatic, but symptoms can include dysphagia, pain on swallowing, and pharyngeal discomfort. The causes are chronic inflammation due to acid secretion and ulceration when mural cells are located in the EGM.^1^

Most primary esophageal adenocarcinomas are derived from the Barrett epithelium of the distal esophagus, and adenocarcinoma arising from the EGM of the proximal esophagus is extremely rare. Here, we describe recurrent esophageal adenocarcinoma arising from the original site in the EGM at the boundary of the cervical and thoracic esophagus in a male patient. He was treated by three endoscopic submucosal dissections (ESDs) and an eventual subtotal esophagectomy.

## CASE REPORT

**FIGURE 1:**
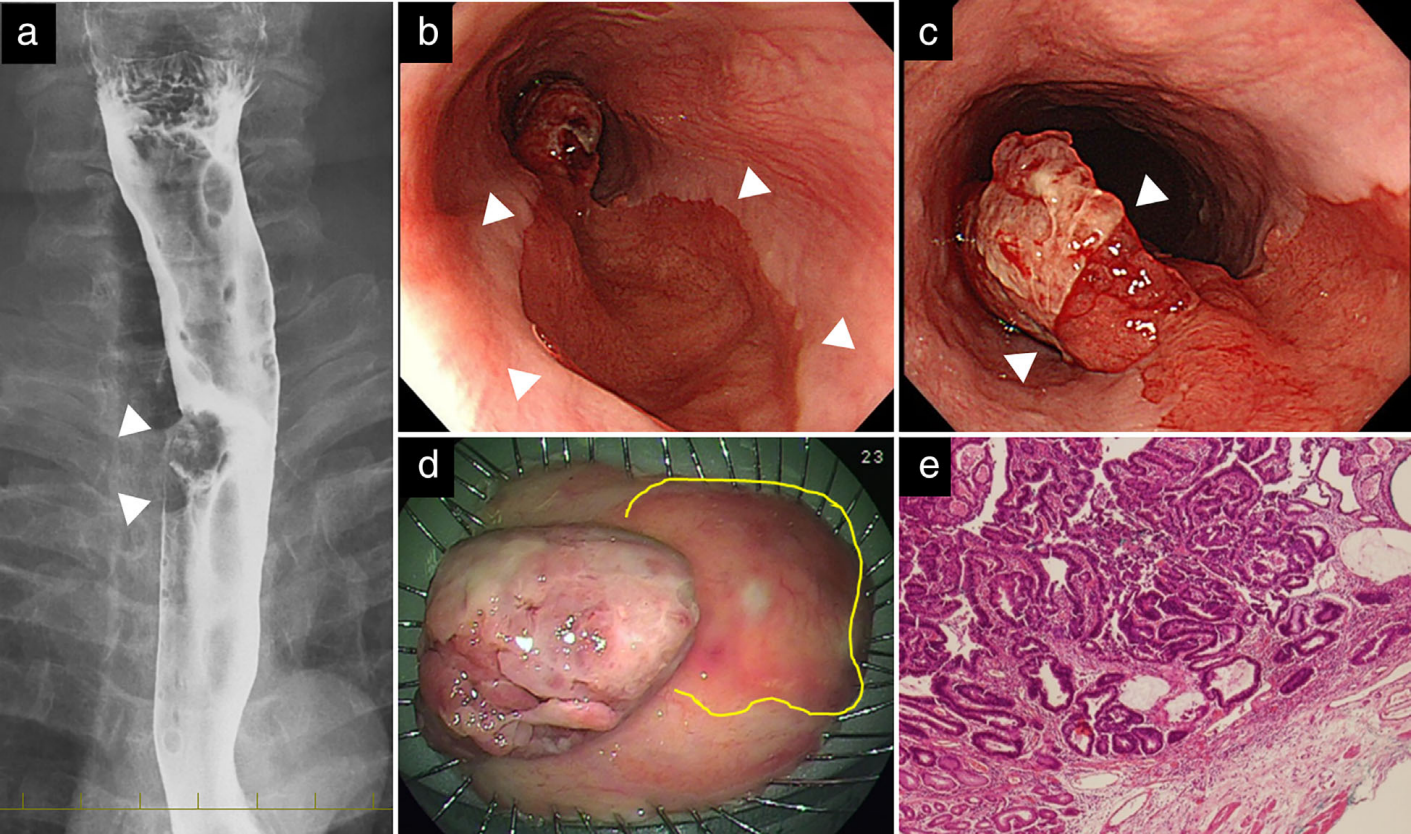
(a) Esophagogram at the initial endoscopic submucosal dissection (ESD) shows an irregularly shaped elevated lesion (20 mm in diameter) at the boundary between the cervical and thoracic esophagus. (b) Esophagogastroduodenoscopy reveals ectopic gastric mucosa (EGM) 18 cm from the incisor row. (c) Stalked elevated lesion arising from EGM. (d) Elevated lesion resected en bloc, including EGM (yellow line). (e) Histologically well‐differentiated adenocarcinoma arising from EGM

A 52‐year‐old man was referred with an esophageal tumor. He had a 70‐pack year smoking history and a history of alcohol abuse, but his family history and medical records were negative for hereditary diseases. The physical and laboratory findings were unremarkable. Esophagography and esophagogastroduodenoscopy revealed an EGM with a stalked elevated lesion (20 mm in diameter) at the boundary of the cervical and thoracic esophagus that was diagnosed as mucosal cancer by endoscopic ultrasound (EUS) (Figure 1(a)–(c)). A biopsy specimen of this lesion was pathologically diagnosed as well‐differentiated adenocarcinoma. Neither lymph nodes nor distant metastases were indicated by computed tomography (CT) and positron‐emission tomography (PET). Based on these findings, the patient was diagnosed with esophageal mucosal adenocarcinoma, cT1a N0 M0 Stage I based on the TNM Classification of Malignant Tumors, 8th edition.^4^ Thus, he was treated by ESD, and the entire tumor lesion including the surrounding EGM was resected en bloc as widely as possible. The lesion was pathologically diagnosed as well‐differentiated adenocarcinoma arising from the EGM and extending up to the muscularis mucosae (MM) and was negative for lateral and vertical resection margins. Therefore, he was judged as being likely to benefit from curative resection (Figure 1(d),(e)).

**FIGURE 2:**
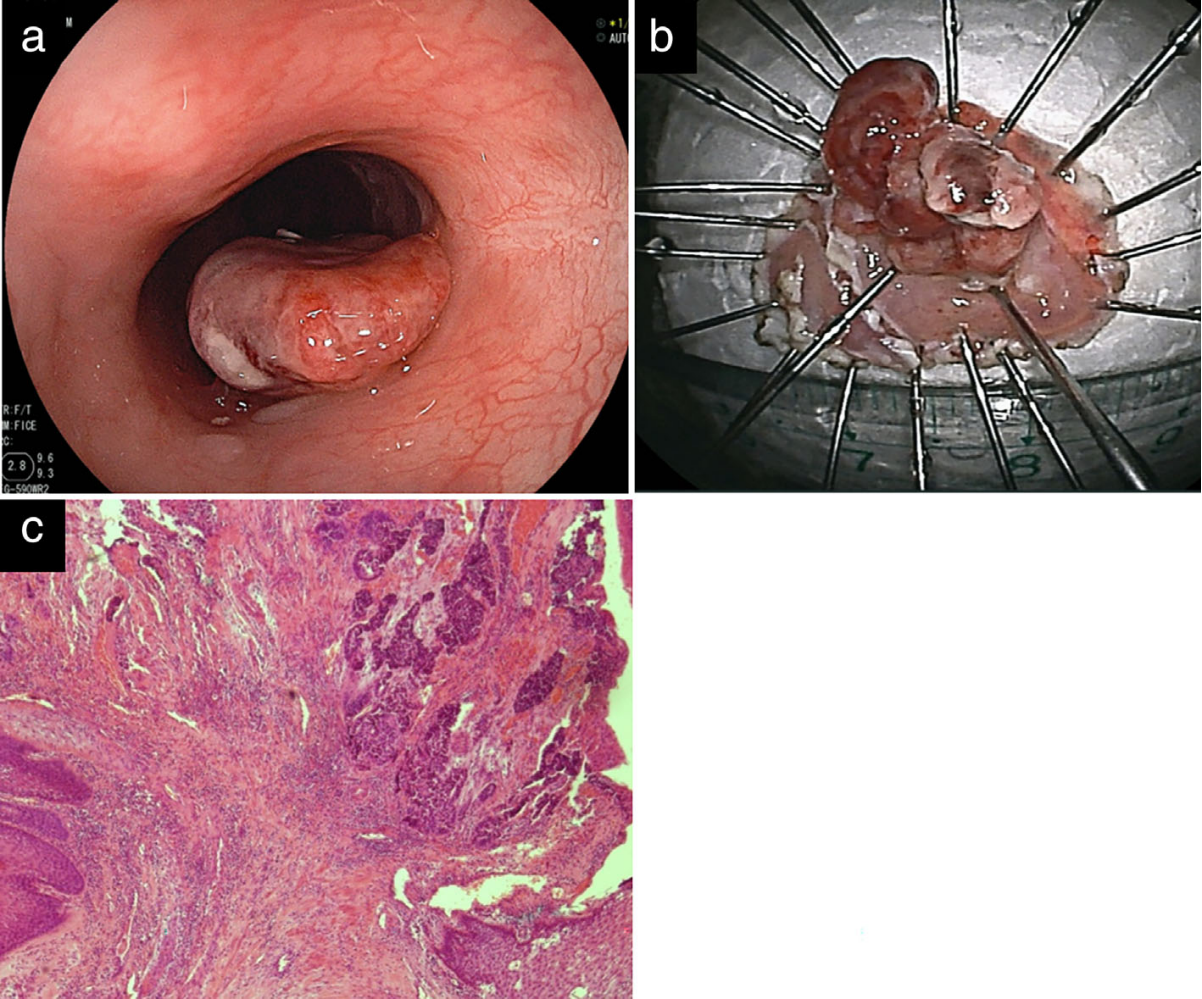
(a) Esophagogastroduodenoscopy at the second endoscopic submucosal dissection (ESD) reveals an elevated stalked lesion (diameter 15 mm) arising from the anal margin of the post‐ESD scar. (b) Specimen resected at second ESD. (c) Histological findings show a moderately differentiated adenocarcinoma arising from the previous ESD scar

The patient was followed up every 6 months thereafter and remained recurrence‐free for 21 months, when a 15‐mm stalked elevated lesion arising from the post‐ESD scar was pathologically diagnosed from a biopsy as adenocarcinoma (Figure 2(a)). The lesion was again diagnosed by EUS, CT, and PET as esophageal mucosal adenocarcinoma without lymph nodes and distant metastasis (cT1a N0 M0 Stage I). Therefore, a second ESD and curative resection including the entire tumor lesion proceeded. This lesion was pathologically diagnosed as moderately differentiated adenocarcinoma up to the MM and was negative for lateral and vertical resection margins (Figure 2(b),(c)).

**FIGURE 3:**
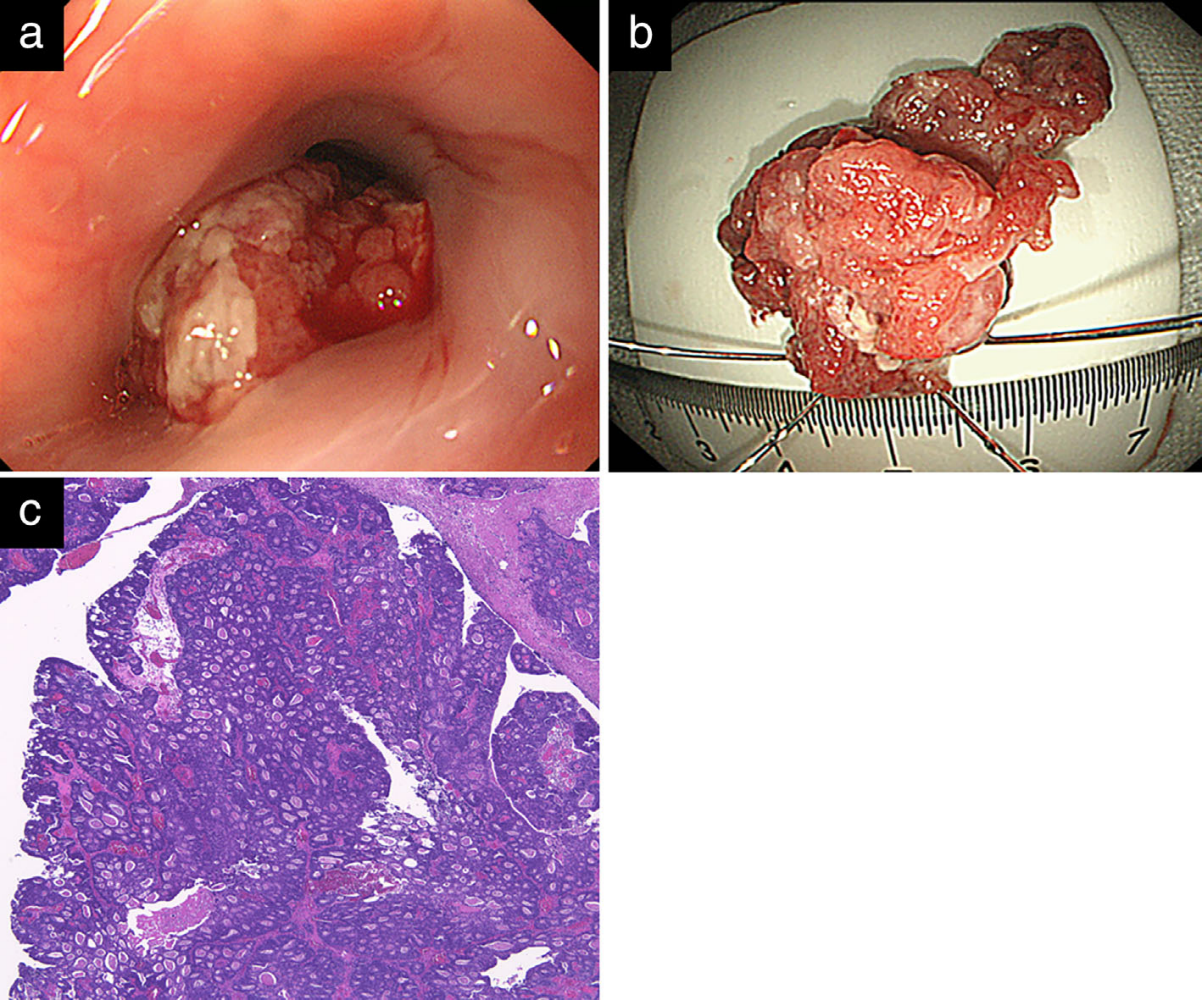
(a) Esophagogastroduodenoscopy at the third endoscopic submucosal dissection (ESD) reveals a 20‐mm stalked elevated lesion arising from the ESD scar. (b) Resected specimen at third ESD. (c) Histological findings show a moderately differentiated adenocarcinoma arising from the previous ESD scar

Stalked elevated lesions (20 mm in diameter) developed twice from the ESD scar 19 months after the second ESD, and this was again pathologically diagnosed as adenocarcinoma by biopsy (Figure 3(a)). Esophageal mucosal adenocarcinoma without lymph node and distant metastasis was also diagnosed (cT1a N0 M0 Stage I). Despite difficulties with debriding the tumor due to repeated post‐ESD scarring, the patient underwent a third ESD en bloc. The resected tumor was pathologically diagnosed as moderately differentiated adenocarcinoma disseminating upwards to the MM with suspicious positive vertical and horizontal resected margins and positive lymphatic invasion (Figure 3(b),(c)). The patient was then referred to our surgical department because the resection was noncurative due to the positive margins and positive lymphatic invasion.

An ESD scar was located at the boundary of the cervical and thoracic esophagus, indicating that the larynx could be preserved. The patient underwent thoracoscopic subtotal esophagectomy with cervical and mediastinal lymph node dissection, and the esophagus was reconstructed using a gastric tube. Postoperative complications did not develop. A postoperative pathological examination revealed no tumor remnants and no lymph node metastasis. The patient remains alive, without signs of cancer recurrence at 5 years after the last surgical procedure.

## DISCUSSION

The incidence of adenocarcinoma among patients with cervical EGM is 0–1.56%.^5^ Orosey et al. reported that among five (1.3%) patients with EGM among 398 esophageal adenocarcinomas diagnosed over 14 years, only 3 (0.8%) had EGM within the proximal esophagus.^6^ Only 58 esophageal adenocarcinomas and EGM were reported between 1950 and 2015 worldwide, and most were from Japan.^7^ The pathogenesis of adenocarcinoma within an EGM might comprise a metaplastic‐dysplastic pathway leading to intestinal metaplasia and an intestinal‐type adenocarcinoma, or the development of adenocarcinoma within gastric/foveolar cells in an EGM.^7^

Among the patients, 53 (91%) were male and 33 (57%) of 58 esophageal adenocarcinomas arising within the EGM were managed by surgical resection, whereas small carcinomas confined to the submucosal surface in 7 (12%) were managed more recently by endoscopic resection.^7^ The treatment strategy for esophageal cancer derived from EGM generally seems to be curative according to the tumor staging, as in common esophageal cancer.^6^ Our patient was clinically diagnosed three times with mucosal adenocarcinoma arising from the same original site with EGM at the boundary of the cervical and thoracic esophagus, without lymph nodes and distant metastasis. Therefore, he was treated three times by ESD with curative intent.

The adenocarcinoma originating from EGM of the proximal esophagus in our patient recurred twice at the same site after ESDs. This might have been due to either multiple occurrences of cancer arising from residual EGM or residual tumor cells proliferating after ESD. Although the EGM was resected as widely as possible at the first ESD, some remained that was detected in a biopsy specimen at follow‐up after ESD. Furthermore, two resected margins after the first two ESDs were pathologically negative for cancer. Therefore, we considered that the repeated lesions might be metachronous esophageal adenocarcinomas that developed from the EGM.

In contrast, one patient has been described with recurrence caused by tumor cells implanted in a mucosal laceration even after curative gastric ESD.^8^ Thus, the possibility that tumor implantation caused the recurrent adenocarcinoma in our patient even after repeated curative ESD cannot be completely ruled out. We could not completely distinguish whether the tumor comprised pathologically metachronous multiple cancers that repeatedly developed from the EGM or recurrent adenocarcinoma due to tumor implantation.

We described an extremely rare recurrent adenocarcinoma originating from EGM of the proximal esophagus. Although the mechanism of repeated tumor development in our patient was difficult to confirm, this experience suggests that at least cancerization from EGM should be considered during periodic follow‐up, even if the risk for cancer is low. Furthermore, early adenocarcinoma originating from the EGM should be treated by complete endoscopic resection, including the entire EGM.

## CONFLICT OF INTEREST

None of the authors has any specific financial interests, relationships or affiliations relevant to the subject of this manuscript.
